# Ultrafast spin dynamics and switching via spin transfer torque in antiferromagnets with weak ferromagnetism

**DOI:** 10.1038/srep35077

**Published:** 2016-10-07

**Authors:** Tae Heon Kim, Peter Grünberg, Song Hee Han, Beongki Cho

**Affiliations:** 1 School of Materials Science and Engineering, Gwangju Institute of Science and Technology (GIST), Gwangju 61005, Republic of Korea; 2 Grünberg Center for Magnetic Nanomaterials, Gwangju Institute of Science and Technology (GIST), Gwangju 61005, Republic of Korea; 3 Division of Navigation Science, Mokpo National University, Mokpo 58628, Republic of Korea

## Abstract

The spin-torque driven dynamics of antiferromagnets with Dzyaloshinskii-Moriya interaction (DMI) were investigated based on the Landau-Lifshitz-Gilbert-Slonczewski equation with antiferromagnetic and ferromagnetic order parameters (***l*** and ***m***, respectively). We demonstrate that antiferromagnets including DMI can be described by a 2-dimensional pendulum model of ***l***. Because ***m*** is coupled with ***l***, together with DMI and exchange energy, close examination of ***m*** provides fundamental understanding of its dynamics in linear and nonlinear regimes. Furthermore, we discuss magnetization reversal as a function of DMI and anisotropy energy induced by a spin current pulse.

Since the first discovery of sub-picosecond demagnetization of ferromagnetic nickel film using femtosecond infrared lasers, ultrafast manipulation of magnetization has raised much interest in terms of both condensed matter physics and applications in information storage devices^1^. Together with the development of femtosecond lasers, a considerable number of research studies have been conducted to explore the microscopic dynamics experimentally as well as theoretically for various magnetic systems^2^,^3^,^4^,^5^,^6^,^7^,^8^,^9^,^10^,^11^,^12^,^13^.

The antiferromagnet (AF) system is a promising structure for ultrafast processes because it has a relatively strong exchange interaction that shifts the precession frequency into the terahertz range. The AF system can be excited or switched at picosecond timescales (significantly faster than ferromagnetic precession^14^,^15^,^16^,^17^), and AF switching through current-induced spin transfer torque has been recently measured electrically^18^.

AF systems with weak ferromagnetism (AWF) might be useful for memory devices because of their weak ferromagnetism and selectively controllable excitation modes^19^. The weak ferromagnetism is associated with broken inversion symmetry in the material and is independent of any ferromagnetic impurities^20^. This type of magnetism has been studied experimentally in the rare earth orthoferrites^21^,^22^,^23^,^24^,^25^,^26^ and rhombohedral antiferromagnet FeBO_3_^27^,^28^ by many research groups. However, analytical approaches to describe AWF dynamics are rare except for AF cases^15^,^29^.

This paper shows that AWF dynamics is governed by the classical pendulum equations on the antiferromagnetic order parameter (***l***), similar to the simple AF case^14^. We demonstrate quantitatively that the occurrence of the second harmonic of the ferromagnetic order parameter (***m***) is direct evidence for a nonlinear regime, including resonant frequency softening^30^, and that the ellipticity of the precessional motion of ***m*** determines the Dzyaloshinskii-Moriya interaction (DMI) energy. Additionally, we propose that sub-lattice dynamics (***s***_1_, ***s***_2_) can be revealed experimentally. We discuss switching efficiency as a function of anisotropy, DMI energy and damping constant (*α*) using spin current pulse with various durations and densities.

## Theory

### AWF dynamics

**Figure 1:**
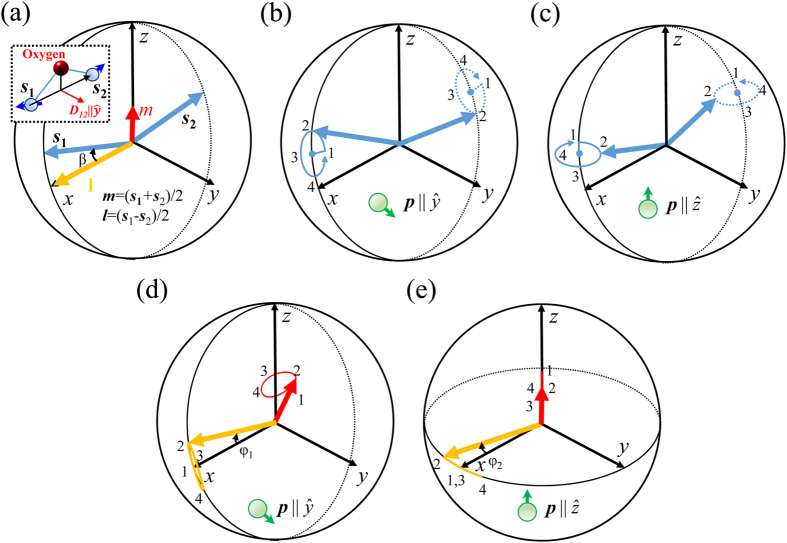
(**a**) Schematic diagram for an antiferromagnet with Dzyaloshinskii-Moriya interaction at *T* < *T*_N_. The ferromagnetic order parameter, *m*, and the antiferromagnetic order parameter, *l*, are defined as (*s*_1_ + *s*_2_)/2 and (*s*_1_ − *s*_2_)/2, respectively. *m* is exaggerated compared to *l*. Inset shows the DM interaction mechanism between the sub-lattices and oxygen. Two resonant modes are selectively excited depending on the injected spin polarization: Sigma mode in *p*||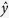 (**b**) and Gamma mode in *p*||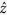 (**c**). (**d,e**) are reproductions of (**b,c**), respectively, in terms of *l* and *m*.

Figure 1 shows AWF static and dynamic configurations based on two sub-lattice models below the Néel temperature^31^. Antiferromagnetically coupled spins lie along the *x*-axis because the anisotropy of the spins occurs along the uniaxial direction, with the magnetic easy axis parallel to *x*-axis, and the spins are tilted along the *z*-axis due to the DMI vector, 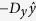, as shown in Fig. 1(a). The DMI produces two resonant modes, called the Sigma mode and the Gamma mode (S- and G-mode, respectively)^19^.

### Landau-Lifshitz-Gilbert-Slonczewski equation

To better understand the kinetics of AWF, the total energy based on two sub-lattices with *i* = 1, 2 is expressed as


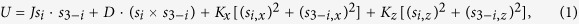


where the normalized magnetization, ***s***_i_ = ***S***_i_/*S*_0_ with *S*_0_ = |***S***_i_| is dimensionless, and *ħ* is the reduced Plank constant. The first term is related to the exchange energy, where *J* is the nearest-neighbor symmetric exchange constant, with the positive sign accounting for AF coupling. The second term describes Dzyaloshinskii-Moriya (DM) energy, where the DM vector, ***D***, is 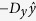, *D*_*y*_ > 0, and its magnitude is relatively weak. The third and fourth terms are anisotropy energies where anisotropy constants are *K*_x_ > 0 and *K*_z_ < 0, indicating magnetic in-plane and out-of-plane anisotropy, respectively. These energy combinations cause the anti-parallel spins to be tilted slightly along the *z*-axis. The dynamics can be described by the coupled Landau-Lifshitz-Gilbert-Slonczewski equation of motion:


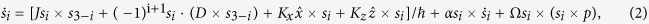


The fifth term is phenomenological damping, which is characterized by the Gilbert damping constant (*α*). The final term is the Slonczewski-type spin transfer torque (STT), where ***p*** is the unit vector of spin polarization, and *Ω* is the STT strength with angular frequency unit, defined as *εħγJ*_*s*_/(2*VS*_0_e), which is proportional to the spin current density, *J*_s_, where *ε* and *V* are the scattering efficiency and volume of AWF region, respectively^14^,^29^,^32^.

We use staggered magnetization, ***l*** = (***s***_1_ − ***s***_2_)/2, and weak magnetization, ***m*** = (***s***_1_ + ***s***_2_)/2, so that Eq. (2) becomes


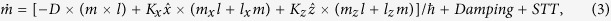



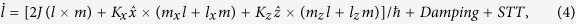


where *Damping* and *STT* are ***α***(***m*** × ***l*** + ***l*** × ***m***) and **Ω[*****m*** × (***l*** × ***p*****) **+ ***l*** × (***m*** × ***p***)], respectively. Equations (3) and (4) are constrained by


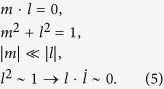


Consider the S-mode excited by spin current with polarization, ***p***||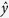. When the STT turns on, ***s***_1_ and ***s***_2_ are dragged slightly toward the *y*-axis by the exchange coupling between the conduction electrons and the magnetic moments, as shown in Fig. 1(b). Consequently, as *m*_*y*_ increases in magnitude, ***l*** is moved away from its equilibrium position. After the STT turns off, ***l*** and ***m*** are subject to an internal magnetic field torque, and ***m*** precesses on the *xy*-plane and fluctuates along the *z*-axis, as shown in Fig. 1(d). (In a simple AF, only *m*_*y*_ fluctuation is shown^14^,^17^,^29^.) This is ascribed to the DMI, coupled with *m*_*x*_ and *m*_*z*_, which causes elliptical polarization of precessional motion of***m***, as shown in Fig. 1(d). The details are discussed below, in conjunction with the second harmonic oscillation of *m*_*z*_.

Because the *l*_*y*_ component is much smaller than *l*_*x*_ and *l*_*z*_, the dynamics of ***l***can be regarded as approximately 2-dimensional (2D) pendulum motion oscillating with angle *φ*_1_ on the *xz*-plane (see Supplementary information). Therefore, we expand Eqs (3) and (4) by using the effective vectors (*l*_*x*_, 0, *l*_*z*_) and (0, *m*_*y*_, 0), and take the cross product of ***l*** on Eq. (4) to extract only ***m***,


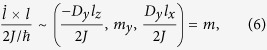



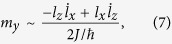


where the equations are simplified by employing Eq. (5) and ignoring terms coupled with anisotropy energy because |*K*_x_*|* and |*K*_z_*|* ≪ *D*_y_ and *J*. Substituting Eq. (7) into Eq. (3), we have the 2D pendulum equations,


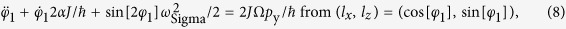


where 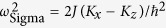.

In G-mode, ***p***||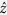; thus, ***s***_1_ and ***s***_2_ are dragged slightly toward the *z*-axis, as shown in Fig. 1(c). As *m*_*z*_ increases in magnitude, with *m*_*x*_ and *m*_*y*_ remaining zero, ***l*** is moved away from the equilibrium position (see Supplementary information). After STT turns off, *m*_*z*_ shows only fluctuation motion and ***l***oscillates on the *xy*-plane, as shown in Fig. 1(e). The methodology in S-mode is used again, so that


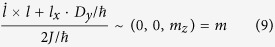



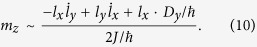


Substituting Eq. (9) into Eq. (3), the 2D pendulum equation becomes


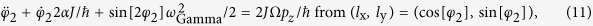


where 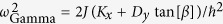, with spin canting angle *β* in equilibrium (l and *l*_*z*_ = 0) because tan[*β*] = *m*_*z*_/*l*_*x*_ = *D*_*y*_/(2*J*). These outcomes confirm White *et al*.^19^.

## Results and Discussion

### Ultrafast dynamics in the terahertz regime

**Figure 2:**
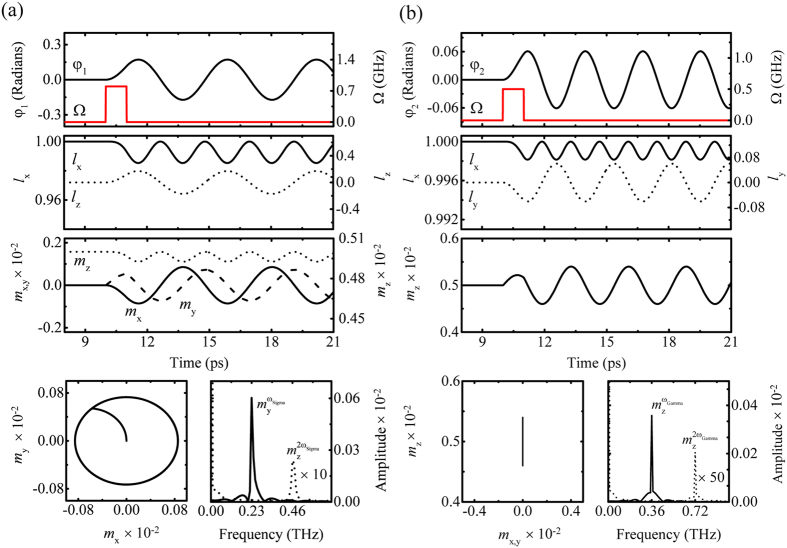
Excitation modes when (**a**) ***p***||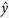 and Ω = 0.8 GHz and (**d**) ***p***||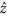 and Ω = 0.5 GHz. Spin trajectories (left) and spectra (right) are shown in fourth row. In both cases, the pulse duration (τ) is 1 ps and the Gilbert damping constant is 0 (see Supplementary Movie 1 and 2).

We introduce the DMI or antisymmetric super-exchange interaction from the triangle spanned among three ions (magnetic ions and oxygen ion). Such an interaction was discovered in the interface between AF and ferromagnet^33^, and between AF and ferrimagnet superlattices^34^, as well as bulk crystals^20^,^21^,^22^,^23^,^24^,^25^,^26^. Here, we suppose a two-layer system consisting of two antiferromagnetic oxides, where the interaction between two magnetic ions arranged along the *x*-axis, and the oxygen ion, shifted slightly to the *z*-axis, gives rise to DM vector,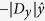, as shown in the inset of Fig. 1(a). Because the magnetic easy axis is the *x*-axis, sub-lattice spins are canted toward the *z*-axis. Additionally, we assume that the magnetic unit cell exhibits weak ferromagnetism, as in the case of rare earth ferrite, ReFeO_3_ single crystal (Re: Er, Tm, and Y, etc). The parameters chosen were *J* = 113.5 meV, *K*_*x*_ = 4.14 *μ*eV, *K*_z_ = 0, and *D*_y_ = 0.01*J* so that spin precession motion is in the terahertz frequency range. To inject spin current into AWF, we exploit the spin hall effect in Pt with strong spin-orbit coupling. Figure 2(a,b) show S-mode and G-mode in AWF (see Supplementary Movie 1 & 2). Moreover, we checked that our analytical results are validated by numerical calculations based on Eq. (1) (see Supplementary Figure 2 and 3). However, for stronger DM energy, we found that the analytical solution deviates from the numerical one because the approximation (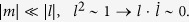) is no longer valid. (see Supplementary information).

### Second harmonic oscillation of *M*_z_ as a nonlinear effect

S- and G-mode dynamics have common characteristic motion: second harmonic oscillations along the *z*-axis. According to Eqs (6) and (9), 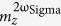 and 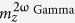 are both responsible for the nonlinearity of *l*_*x*_, together with the resonant frequency softening^30^. For example, *φ*_1_(t) is sinusoidal in *Ω* = 0.8 GHz; as a results, *m*_*z*_ = *l*_*x*_ · *D*_*y*_/*ħ* ∼ cos[*φ*_1_(*t*)] = cos[*A*sin[*ω*_Sigma_*t*]] is replaced with ~(1 − *A*^2^/4) − *A*^2^cos[2*ω*_Sigma_*t*]/4 by its first order Taylor expansion. Likewise, 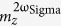
and 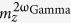
are shown in Fig. 2(a,b), fourth row, right.

### Determination of DMI strength

The DMI strength can be obtained by examining the first harmonic precession on the *xy*-plane in the S-mode, 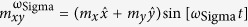. Using Eqs (6) and (7), the ellipticity, *ε* is calculated as *m*_*y*_(*t*)/*m*_*x*_(*t*) = [−2*J*/(*D*_*y*_*l*_*z*_)] *ħ*(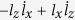)/(2*J*)] = *Aħω*_Sigma_cos[*ω*_Sigma_*t*]/(*D*_*y*_sin[*A*sin[*ω*_Sigma_*t*]]). If we assume *A* is small enough, *ε*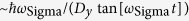. For example, 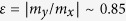 is constant within a few percent with 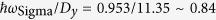, as shown in Fig. 2(a), fourth row, left. Experimentally, the precessional polarization in S-mode can be measured using optical tools: terahertz time domain spectroscopy^23^,^24^,^25^,^26^,^27^,^28^,^30^, or time resolved magneto optical Kerr/Faraday rotation^16^,^21^,^22^.

### *s*_1_ and *s*_2_ deduced from *m* and *l*

Once the DMI strength is determined, *J* is easily estimated by using well-known antisymmetric exchange model, *M*_*s*_ ∼ *M*_0_*D*_*y*_/(2*J*)^35^, where saturation magnetization, *M*_s_ can be measured by using a conventional sample vibrating magnetometer. *M*_0_ is the number of magnetic ions per volume or mole. Conventional time domain terahertz spectroscopy (or time resolved magneto optical Kerr/Faraday rotation technique) can be used to observe ***m***(t). From the Fourier transform of *m*_x_(t) (or *m*_y_(t)) and *m*_z_(t), the resonant frequencies (and thereby *K*_x_ and *K*_z_) are obtained. As ***m***, *D*, and *J* are determined, ***l*** could be estimated using Eqs (6) and (9). For example, in S-mode, *l*_*z*_ and *l*_*x*_ can be deduced from *m*_*x*_and *m*_*z*_ using Eqs (5) and (6). In G-mode, *l*_*x*_ and *l*_z_ can be estimated using Eqs (8) and (9) and the spectral amplitude and phase information. The resulting ***s***_1_ and ***s***_2_ are shown in Fig. 1(b,c). In contrast, *l*_*x*_ is not extractable in simple AF because of the lack of DMI^14^.

### Switching mechanism and efficiency

**Figure 3:**
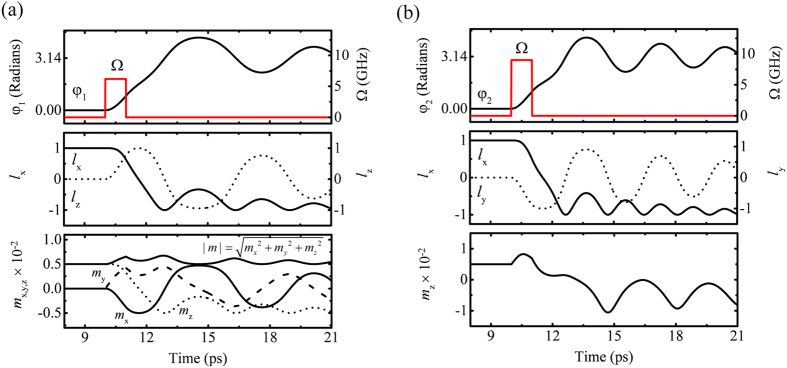
Magnetization switching when (**a**) ***p***||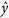 and Ω = 6.2 GHz and (**b**) ***p***||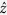 and Ω = 9 GHz. In both cases, the pulse duration (τ) is 1 ps and the Gilbert damping constant is 0.0005 (see Supplementary Movie 3 and 4).

Figure 3(a,b) show the switching process for S-mode and G-mode, respectively (see Supplementary Movie 3 and 4). In S-mode, |***m***|, defined as 
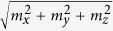
, (or *m*_*z*_ in G-mode), increases in magnitude less than 1% for |***l***_max_| ~ 1 with the spin current pulse. Additional canting is converted into kinetic energy, and if the kinetic energy exceeds the maximum potential energy, spin reversal occurs after the pulse has been turned off. This is inertia-driven switching^17^, and the switching is identically applied in G-mode.

**Figure 4:**
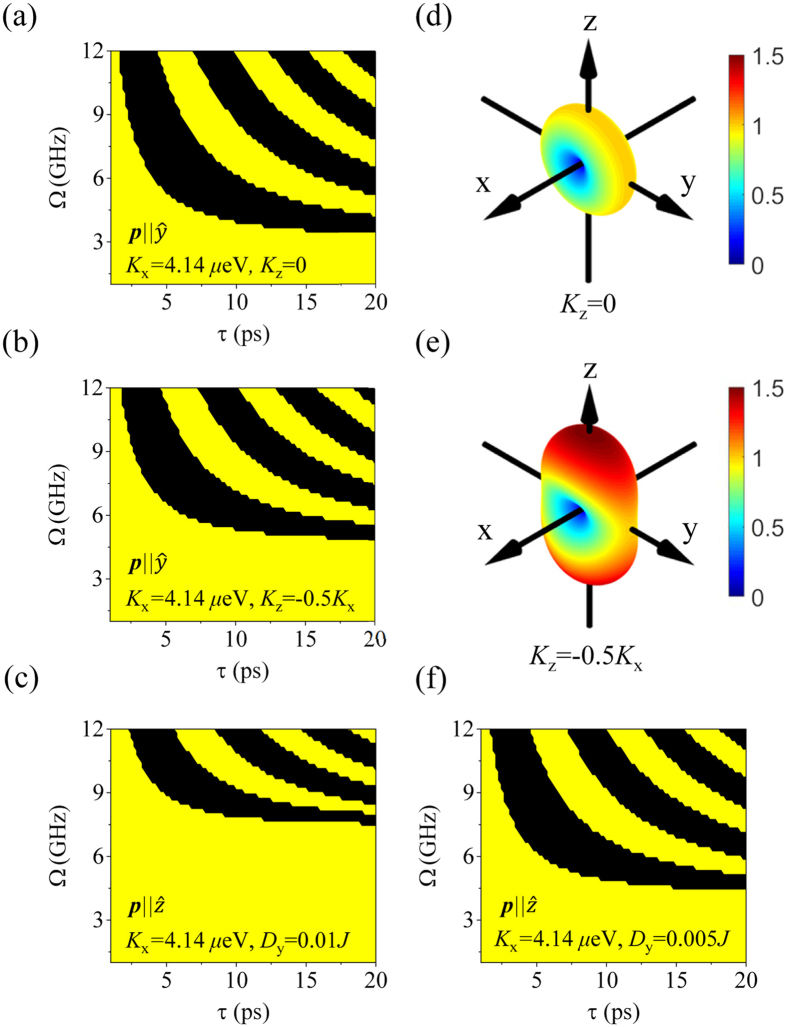
(**a–c**,**f**) Periodic patterns of terminal phase of *l*_x_ for various conditions of *K*_x_, *K*_z_, *D*_y_, and ***p*** in the Ω versus τ plot after excitation by a pulsed spin current. From left down to right up, the terminal phases of *l*_x_ are indicated by nπ, n = 0, 1, 2, … (even = yellow, odd = black), α = 0.01. (**d**–**e**) Potential energy distribution with *K*_z_ = 0, *K*_z_ = −0.5 *K*_x_, and *K*_x_ = 4.14 *μ*eV.

Because either *ω*_Sigma_ or *ω*_Gamma_ could be manipulated by *K*_*z*_ or *D*_*y*_, switching efficiency should be considered for these parameters. Figure 4 shows the periodic patterns for the terminal phase of *l*_*x*_ for various values of *K*_*x*_ and *D*_*y*_ after excitation by spin current pulses for several values of *τ*, *Ω* and ***p***. From left down to right up, terminal phases of *l*_*x*_ are indicated by nπ, n = 0, 1, 2.

**Figure 5:**
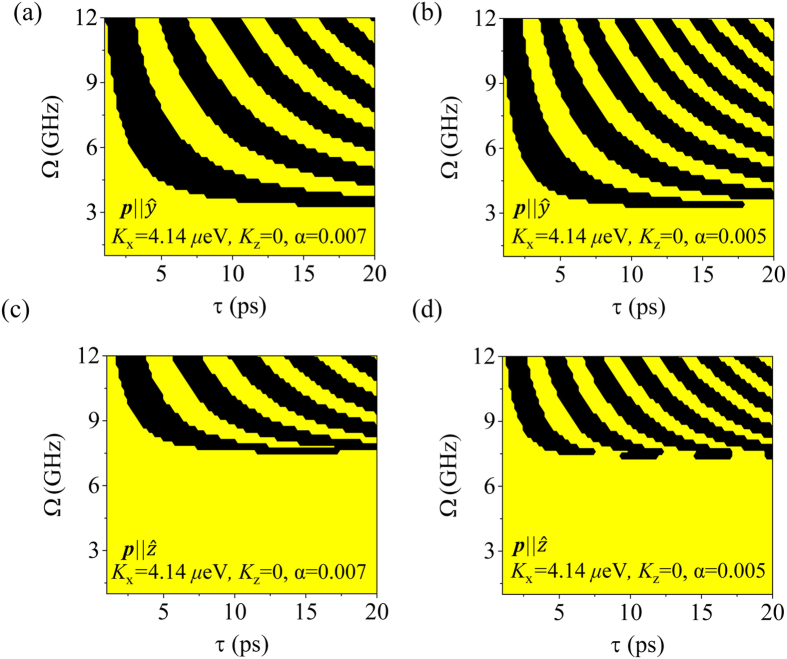
Periodic patterns of the terminal phase of *l*_x_ for different damping constants for S- and G-mode: (**a**,**c**) the damping constant is 0.007, and (**b**,**d**) the damping constant is 0.005. In these cases, the pulse duration is 1 ps.

When the potential barrier increases from *K*_*z*_ = 0 in Fig. 4(d) to *K*_*z*_ = −0.5|*K*_*x*_| in Fig. 4(e), ***l*** in S-mode must overcome the higher potential barrier on the *xz*-plane. Thus, phases of *l*_*x*_ are shifted upward in Fig. 4(b), compared to Fig. 4(a). Another factor to modify the switching efficiency is the DMI strength in G-mode, where *K*_*z*_ does not play a role in the control of the energy barrier on the *xy*-plane because the energy barrier on the *xz*-plane is controllable by *K*_z_. When *D*_*y*_ = 0.01 *J* in Fig. 4(c), the pendulum system energy is higher than that of *D*_*y*_ = 0.005 *J* in Fig. 4(f), and the first switching demands higher STT strength. Experimentally, magnetic materials have temperature dependence on anisotropy energies or thickness dependence on *D*_*y*_^33^,^36^. Additionally, the interface engineering is used to change DMI strength^37^. Applying these properties, one can expect to switch magnetization under optimal conditions. In particular, AWF systems, which have anisotropy with two easy-axes (*K*_*x*_ and *K*_*z*_ > 0), would undergo switching at a lower critical STT strength (*Ω*_c_) in S-mode, because *K*_z_ lowers the switching barrier. Finally, we checked the *α* dependence. When the pulse duration (τ) is short, the damping effect obviously lowers *Ω*_c_: For example, *Ω*_c_ = 4.4 GHz, τ = 5 ps for *α* = 0.007 and *Ω*_c_ = 3.8 GHz, *τ* = 5 ps for *α* = 0.005 in Fig. 5(a,b). In general, as *α* becomes smaller, the periodic patterns become narrower^14^. In particular, in short *τ*, the slope of the phase boundary is steep and dependent on *α*; thus, one might doubt its stable functionality as a device. In long τ, *Ω*_c_ is much reduced and finally become minimized, but its magnitude is not further reduced for variation of *α*.

## Conclusion

In summary, we investigated the process of precession motion in antiferromagnets with weak ferromagnetism through spin transfer torque. Although DMI splits the AF resonant mode into S- and G-modes, the modes are also be interpreted as pendulum models on ***l***. Because ***l*** and DMI energy are coupled and independently extractable through measurement of ***m***, dynamic analysis of ***m*** provides fundamental understanding of sub-lattice dynamics, as shown in Fig. 1(b,c). Adjustment of appropriate parameters, such as the anisotropy barrier and DMI strength, provide more efficient magnetization reversal.

## Additional Information

**How to cite this article**: Kim, T. H. *et al*. Ultrafast spin dynamics and switching via spin transfer torque in antiferromagnets with weak ferromagnetism. *Sci. Rep*. **6**, 35077; doi: 10.1038/srep35077 (2016).

## Supplementary Material

Supplementary Information

Supplementary Movie S1

Supplementary Movie S2

Supplementary Movie S3

Supplementary Movie S4
