# Supplementary material for: Ultrafast spin dynamics and switching via spin transfer torque in antiferromagnets with weak ferromagnetism
Source: Sci Rep. 2016 Oct 7;6:35077. doi: 10.1038/srep35077 (PMC5054374; doi:10.1038/srep35077)
Supplement: Supplementary Information [file srep35077-s1.pdf]

SUPPLEMENTARY INFORMATION

Ultrafast spin dynamics and switching via spin transfer torque in antiferromagnets with weak ferromagnetism

Tae Heon Kim[1,2], Peter Gruenberg[2], Song Hee Han[3], and Beongki Cho[1,2,a)]

[1]*School of Materials Science and Engineering, Gwangju Institute of Science and Technology (GIST), Gwangju 61005, Republic of Korea*

[2]*Gruenberg Center for Magnetic Nanomaterials, Gwangju Institute of Science and Technology (GIST), Gwangju 61005, Republic of Korea*

[3]*Division of Navigation Science, Mokpo National University, Mokpo 58628, Republic of Korea*

[a)] Correspondence and requests for materials should be addressed to B. K. Cho.

(chobk@gist.ac.kr).

We present here :

1. Microscopic understanding for two resonant modes
2. Analytical solution versus numerical solution

   - Spin dynamics at various DMI energies (D = 0.01 J, 0.1 J)

3. Movies of the numerical solutions of the magnetization dynamics in terms of

   - Sub-lattice dynamics, $\boldsymbol{s}_1$**(t)** and, $\boldsymbol{s}_2$**(t)**
   - Staggered and weak magnetization dynamics, $\boldsymbol{l}$**(t)** and, $\boldsymbol{m}$**(t)**

Microscopic understanding for resonant modes

First, it is helpful to understand simple antiferromagnets without Dzyaloshinskii-Moriya (DM) interaction. Assume that antiferromagnetically coupled two sub-lattices lie along the x-axis due to uniaxial anisotropy parallel to the x-axis (easy axis). After tipping toward the y-axis by spin transfer torque, sub-lattices are in precession motion along anisotropic field direction parallel to the x- or - x-axis for $\boldsymbol{s}_1$ or $\boldsymbol{s}_2$, respectively. As a result, the x and z components of the sub-lattices are out of phase with respect to each other and thereby are cancelled out (because the two motions are symmetric), whereas the y-components are in phase and reinforced, as shown in Fig. S1(a-II). Therefore, m_y exhibits fluctuating motion, as shown in Fig. S1(a-I). Likewise, if spin torque is applied to the z-axis, the fluctuation of m_z occurs, because simple antiferromagnet is magnetically symmetric to the y- or z-axis. When we consider sub-lattices dynamics including DM interaction, the unique magnetically symmetric axis is the z-axis, and, thereby, they exhibits symmetric motion in G-mode, as shown in Fig. 1(c) in the article. All components are cancelled out except for m_z, which exhibits fluctuating motion similar to a simple AF. Next, consider the S-mode. As shown in Fig. 1(b) or Fig. S1(b-II), spins are in precession motion along the equilibrium position that is not perfectly parallel to the x-axis because of spin canting. Therefore, the x- and z-component of the sub-lattices are not cancelled out, resulting in non-zero m_x and m_z components. This leads the precession of m_{xy} and fluctuation of m_z.

Spin dynamics in various DMI energies (*D* = 0.01 *J*, 0.1 *J*)

Figures S2 and S4 show the analytical and numerical results when $D_y = 0.01\,J$ and $0.1\,J$. As the ratio of D_y/J becomes larger, or even equal to 1, the approximation discussed in analytical consideration ($|m| << |l|,\ l^2 \sim 1 \rightarrow l \cdot \dot{l} \sim 0.$) becomes invalid anymore, which is shown in Fig. S4, because a larger canting angle, β, and, thereby $|\boldsymbol{m}|$, is expected. However, two equations of motion, Eqs. (6) and (10), enable understanding of the tendency of the numerically calculated dynamics for various D_y/J . Compared to $D_y = 0.01\,J$, m_x and m_z increased by 10 times, but there is almost no change in m_y for $D_y = 0.1\,J$, as shown in Fig. S3. This behavior could be understood from equation (6): $m \sim (\frac{-D_y l_z}{2J}, \frac{-l_z \dot{l}_x + l_x \dot{l}_z}{2J/\hbar}, \frac{D_y l_x}{2J})$,

$m_y \sim \frac{-l_z\dot{l}_x + l_x\dot{l}_z}{2J/\hbar} = A\hbar\omega_{Sigma}\,\mathrm{Sin}(\omega_{Sigma}t)/(2J)$. Likewise, in G-mode, Equation (10) is expressed as $m_z \sim \frac{-l_x\dot{l}_y + l_y\dot{l}_x + l_x\cdot D_y/\hbar}{2J/\hbar} \sim (-A\hbar\omega_{Gamma}\,\mathrm{Sin}(\omega_{Gamma}t)+D_y)/(2J)$.

Supplementary Figures

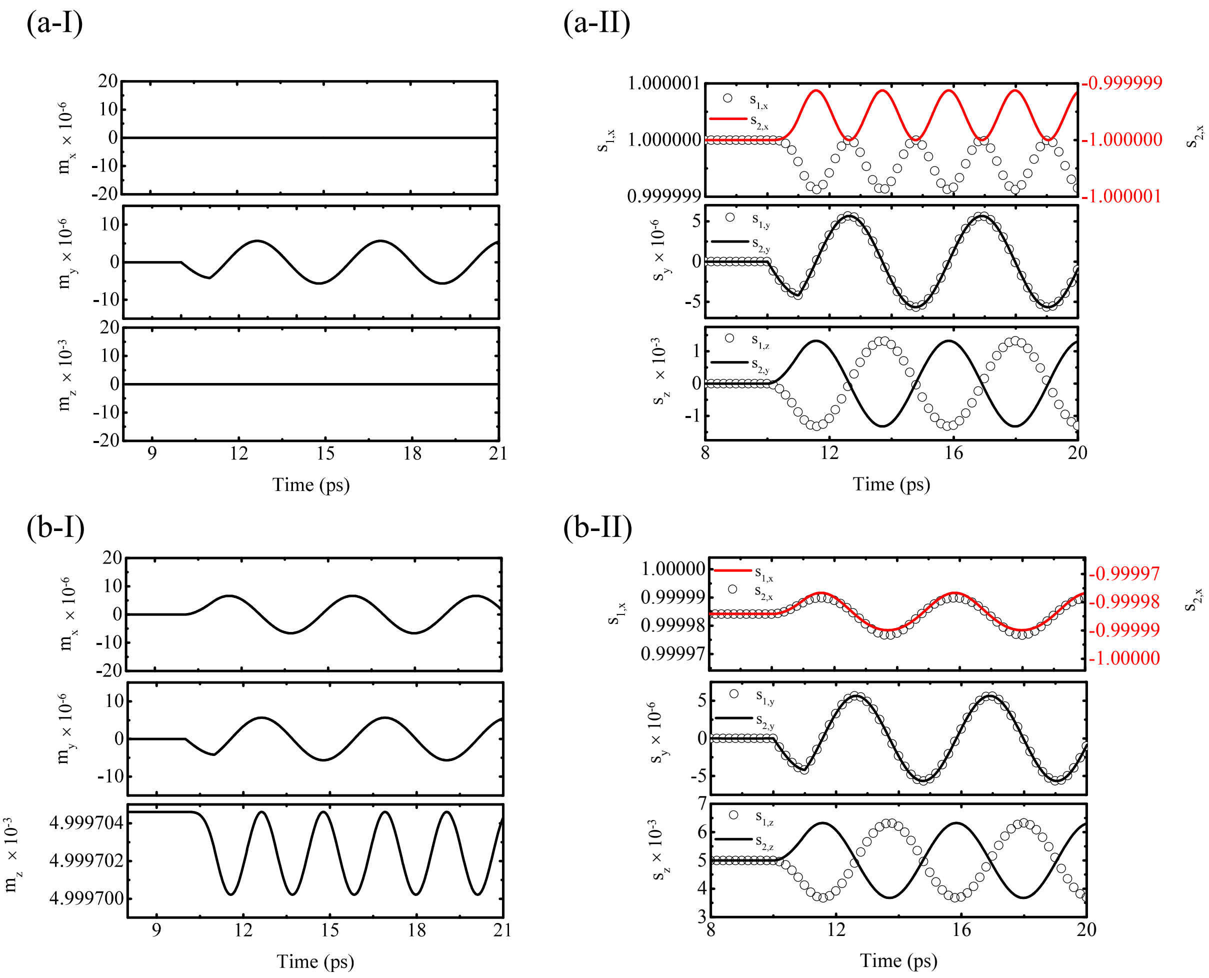

Figure S1. Comparison of resonant modes between a simple antiferromagnet (a-I,II) and an antiferromagnet with weak ferromagnetism (b-I,II). In both cases, the magnetic systems are excited by $\boldsymbol{p} \parallel \hat{y}$, with Ω= 60 GHz, pulse duration = 1 ps, and Gilbert damping constant = 0.

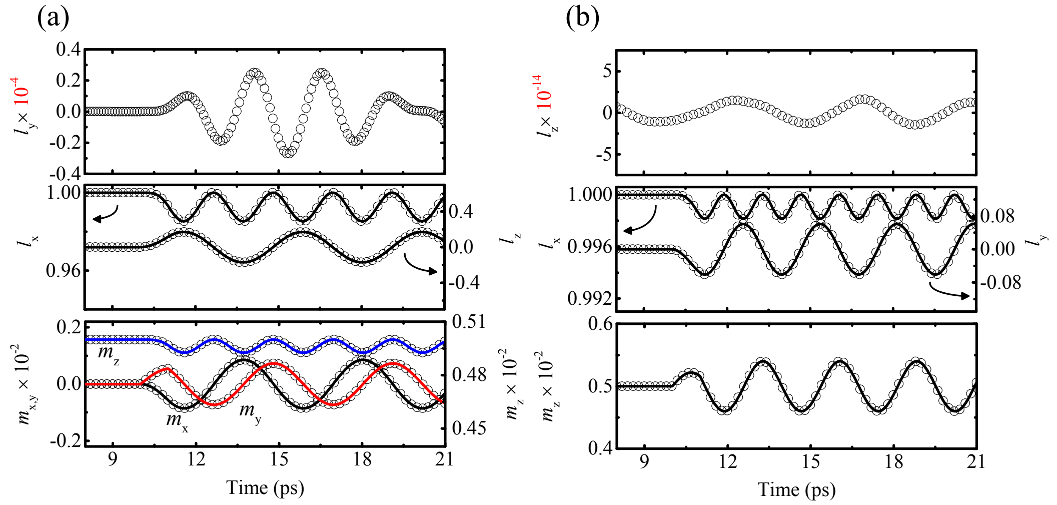

Figure S2. Analytical (solid line) and numerical (open circle) solutions for excitation mode when (a) $\boldsymbol{p} \| \hat{y}$ and Ω= 0.8 GHz and (b) $\boldsymbol{p} \| \hat{z}$ and Ω = 0.5 GHz. Here, l_y in (a) and l_z in (b) is much smaller than the others: In both cases, D_y = 0.01 J, pulse duration = 1 ps and Gilbert damping constant = 0

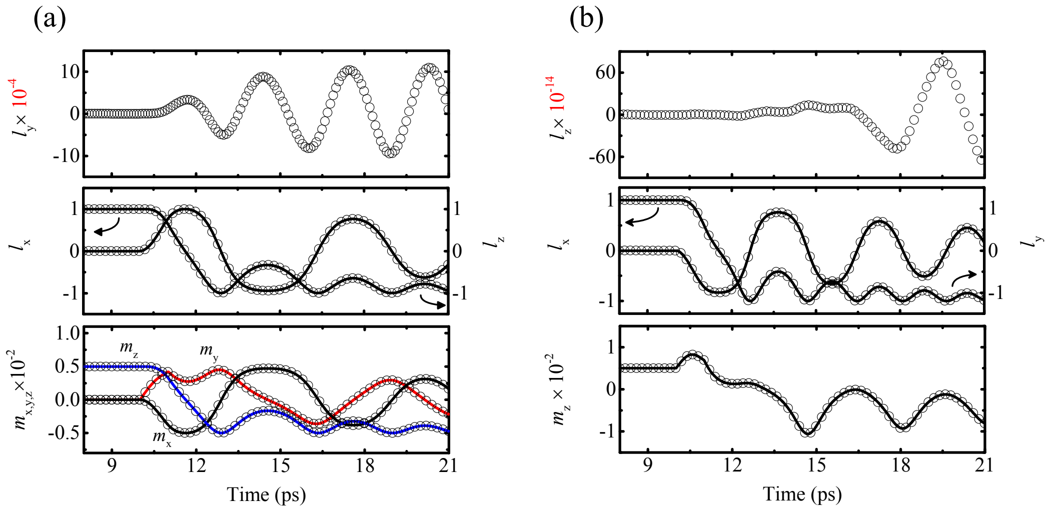

Figure S3. Analytical (solid line) and numerical (open circle) solutions for the switching mode when (a) $\boldsymbol{p} \| \hat{y}$ and Ω= 6.2 GHz and (b) $\boldsymbol{p} \| \hat{z}$ and Ω = 9 GHz. Here, l_y in (a) and l_z in (b) is much smaller than the others: In both cases, D_y = 0.01 J, pulse duration = 1 ps and Gilbert damping constant = 0.1.

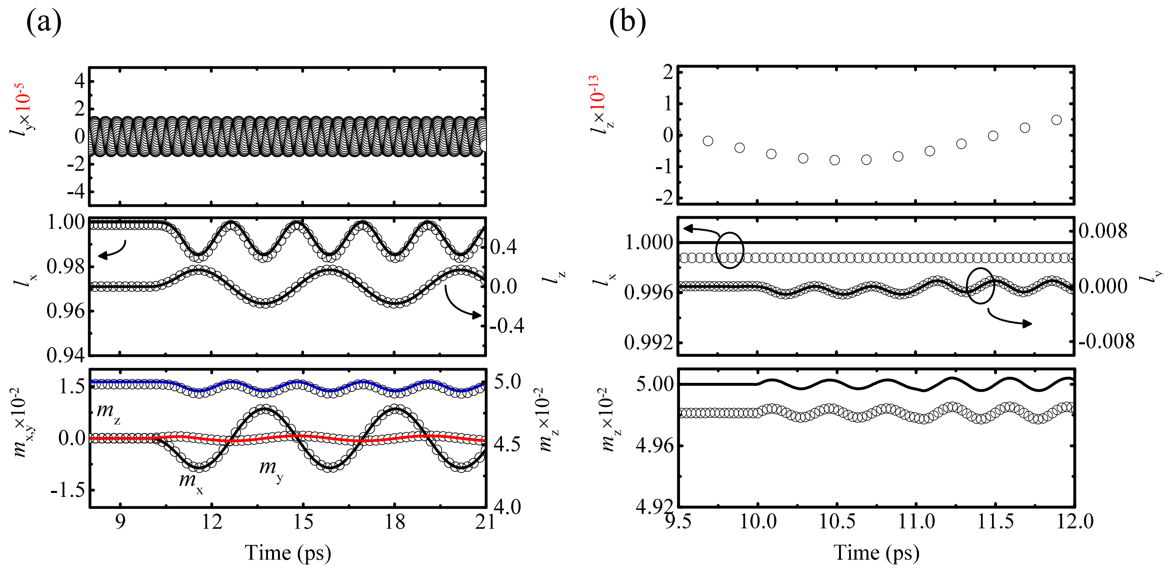

Figure S4. Analytical (solid line) and numerical (open circle) solutions for the switching mode when (a) $\boldsymbol{p} \| \hat{y}$ and Ω= 0.8 GHz and (b) $\boldsymbol{p} \| \hat{z}$ and Ω = 0.5 GHz. Here, l_y in (a) and l_z in (b) is much smaller than the others. In both cases, D_y = 0.1 *J*, pulse duration = 1 ps and Gilbert damping constant = 0.

Supplementary Movie Legends

Supplementary Movie 1. Magnetization excitation when $\boldsymbol{p} \| \hat{y}$ and Ω = 0.8 GHz. The pulse duration is 1 ps and the Gilbert damping constant is 0.

Supplementary Movie 2. Magnetization excitation when $\boldsymbol{p} \| \hat{z}$ and Ω = 0.5 GHz. The pulse duration is 1 ps and the Gilbert damping constant is 0.

Supplementary Movie 3. Magnetization switching when $\boldsymbol{p} \| \hat{y}$ and Ω = 6.2 GHz. The pulse duration is 1 ps and the Gilbert damping constant is 0.0005.

Supplementary Movie 4. Magnetization switching when $\boldsymbol{p} \| \hat{z}$ and Ω = 9 GHz. The pulse

duration is 1 ps and the Gilbert damping constant is 0.0005.
